# Enzyme assays for synthesis and degradation of 2-5As and other 2′-5′ oligonucleotides

**DOI:** 10.1186/s12858-015-0043-8

**Published:** 2015-06-26

**Authors:** Jesper Buchhave Poulsen, Karina Hansen Kjær, Just Justesen, Pia Møller Martensen

**Affiliations:** Department of Molecular Biology and Genetics, Aarhus University, C.F. Møllers Allé 3, DK-8000 Aarhus C, Denmark

**Keywords:** 2′-5′ oligoadenylate synthetase (OAS), 2′-5′ oligoadenylate nuclease, 5′-triphosphorylated, 2′-5′-linked oligoadenylate polyribonucleotides (2-5As), 5′-dephosphorylated forms of 2-5As (2-5A core molecules), Enzyme assays

## Abstract

**Background:**

The 5′-triphosphorylated, 2′-5′-linked oligoadenylate polyribonucleotides (2-5As) are central to the interferon-induced antiviral 2-5A system. The 2-5As bind and activate the RNase L, an endoRNase degrading viral and cellular RNA leading to inhibition of viral replication. The 2-5A system is tightly controlled by synthesis and degradation of 2-5As. Whereas synthesis is mediated by the 2′-5′ oligoadenylate synthetase family of enzymes, degradation seems to be orchestrated by multiple enzyme nucleases including phosphodiesterase 12, the ectonucleotide pyrophosphatase/phosphodiesterase 1 and the A-kinase anchoring protein 7.

**Results:**

Here we present assay tools for identification and characterization of the enzymes regulating cellular 2-5A levels. A procedure is described for the production of 2′-5′ oligoadenylates, which are then used as substrates for development and demonstration of enzyme assays measuring synthetase and nuclease activities, respectively. The synthetase assays produce only a single reaction product allowing for very precise kinetic assessment of the enzymes. We present an assay using dATP and the A(pA)_3_ tetramer core as substrates, which requires prior isolation of A(pA)_3_. A synthetase assay using either of the dNTPs individually together with NAD^+^ as substrates is also presented. The nuclease reactions make use of the isolated 2′-5′ oligoadenylates in producing a mixture of shorter reaction products, which are resolved by ion-exchange chromatography to determine the enzyme activities. A purified human 2′-5′ oligoadenylate synthetase and a purified human phosphodiesterase 12 along with crude extracts expressing those proteins, are used to demonstrate the assays.

**Conclusions:**

This paper comprises an assay toolbox for identification and characterization of the synthetases and nucleases regulating cellular 2-5A levels. Assays are presented for both enzyme families. The assays can also be used to address a broader cellular role of the OAS enzymes, based on the multiple substrate specificity intrinsic to these proteins.

**Electronic supplementary material:**

The online version of this article (doi:10.1186/s12858-015-0043-8) contains supplementary material, which is available to authorized users.

## Background

5′-triphosphorylated, 2′-5′-linked oligoadenylate polyribonucleotides (2-5As) of the structure pppA(pA)_n_ where n ≥ 1, are the key components of the interferon (IFN)-induced antiviral 2-5A system. IFNs are extracellular cytokines specific to (jawed) vertebrates and central to host innate immunity. Upon binding of cognate receptors, they induce numerous intracellular signaling cascades, leading to transcriptional activation of several hundred genes, which altogether determine the biological outcome involving antiviral, antitumoral, and immunomodulatory effects [1–4]. One gene family induced by IFNs is the double-stranded RNA-dependent 2′-5′ oligoadenylate synthetases (dsRNA-dependent OASs) in humans comprising four members, OAS1, OAS2, OAS3 and OAS-like (OASL). At least six OAS isoforms are expressed from these four human genes: two splice variants from the OAS1 and OAS2 genes and a single variant from each of the OASL and OAS3 genes [5, 6]. In comparison, the mice genome encodes multiple OAS genes: eight OAS1 genes, one each of the OAS2 and OAS3 genes and two OASL genes, believed to have arisen from duplication and divergence events from a common ancestral gene [7, 8]. Upon binding of viral dsRNA, the OAS enzymes synthesize 2-5As from ATP leading to accumulation of 2-5As. These 2-5As in turn activate RNase L, an endoRNase degrading viral and cellular RNA. RNase L cleaves RNA 3′ to UN sequences, but mainly at UA and UU sites, N being any of the nucleotide bases in RNA, and this results in inhibition of viral replication [9–12]. Note that despite of binding dsRNA and ATP, the human OASL is catalytically inactive due to key mutations in the active site compared with the other OAS isoforms [13].

2-5A catalysis generally proceeds by hydrolysis of the phosphoanhydride linking the α and β phosphates of ATP when bound in the so-called donor-site of the enzymes resulting in pyrophosphate (PPi) and AMP, which is subsequently linked to the free 2′-OH group of a second ATP molecule or a 2-5A molecule placed in the acceptor-site of the enzymes [6]. The OASs actually combines a broad range of substrates (not only ATP) to form 2′-5′ hetero- oligonucleotides *in vitro*, thus they are better described as general 2′-5′-nucleotidyl transferases [14, 15]. The requirements on the acceptor-site is a 2′-end positioned AMP linked in an RpA configuration, where R specifies either ATP, nicotinamide adenine dinucleotide (NAD^+^), or oligomeric or polymeric primers (*e.g.* tRNA, Ap_4_A, Ap_3_A). In the donor-site, all nucleoside-triphosphates (NTPs) as well as deoxy-nucleoside-triphosphates (dNTPs) can be used, N specifying the nucleotide bases in DNA and RNA. The following general reaction scheme apply to the varied enzyme capabilities of the OASs: *RpA + (d)NTP → PPi + RpA-(d)NMP* in which the incorporation of AMP prompts for oligoadenylate synthesis by means of multiple 2′ elongation events (the other NMPs and dNMPs make up single incorporation events only).

Due to low sequence specificity, RNase L degrades cellular RNA and prolonged activation results in an anti-proliferative response leading to apoptosis [9–11, 16]. Immediate de-activation of RNase L is therefore required following viral infection, which can be achieved by either of two ways, (*i*) through the reversible binding of an RNase L inhibitor allowing for temporary inactivation, or (*ii*) by degradation of the activator 2-5As leading to persistent inactivation [17, 18]. The identification and exploration of cellular nucleases degrading 2-5As and 2-5A core molecules (the 5′-dephosphorylated forms of 2-5As), is currently ongoing. At present, three enzyme candidates exist: the phosphodiesterase 12 (PDE12) [18], the ectonucleotide pyrophosphatase/phosphodiesterase 1 (ENPP1) [19] and the A-kinase anchoring protein 7 (AKAP7) [20]. Even though these enzymes all degrade the 2′-5′ oligoadenylates *in vitro*, the evidence that they truly antagonize the 2-5A system in cells has yet to be reported. Also two viral proteins, ns2 [21] and VP3 [22] belonging to the group 2a betacoronaviruses and group A rotaviruses, respectively, have been found to degrade the 2′-5′ oligoadenylates *in vitro*. In addition, the ns2 and VP3 proteins have been shown to antagonize the 2-5A pathway during viral infection by preventing RNase L activation, thereby evading the host innate immune response [21, 22]. PDE12 is localized in the mitochondrial matrix [23], ENPP1 in the plasma membrane with its active site pointing towards the extracellular matrix [24] and AKAP7 in the nucleus of cells [25]. 2-5As are mainly produced in the cytosol (and to some extend in the nucleus), which is also the site of viral replication [26–29]. For PDE12 and ENPP1, the 2′-5′ oligoadenylates therefore need to traverse a membrane, *i.e.* either the inner mitochondrial membrane or the plasma membrane in order for degradation to take place. How exactly this would occur remains unknown, though processes like receptor-mediated transport or diffusion may be involved. By contrast, AKAP7 may have access to the 2′-5′ oligoadenylates based on its cellular localization and the fact that nuclear pores allow for diffusion of molecules between compartments. The ability of AKAP7 to restore infectivity of a mutant murine coronavirus carrying an inactivated variant of the ns2 gene has recently been investigated [20]. It was found that a mislocalized version of AKAP7 present in the cytoplasm could restore the infectivity of the mutant virus, which was not the case for the full-length protein localized in the nucleus. This suggests that the 2′-5′ nucleases must be localized in very close proximity to the sites of viral replication, in order to maintain tight regulation over the 2-5A system. Also 5′-phosphatases trimming 2-5As to 2-5A core molecules might take play in degradation of 2′-5′ oligoadenylates in cells. This is based on experiments showing accumulation of 2-5A core molecules over 2-5As in cells upon viral infection [30, 31]. We hypothesize that regulation of 2-5As in cells is tightly controlled and orchestrated by multiple nucleases and phosphatases, some of which remains to be identified.

Chemical- as well as enzyme-based strategies have been used to synthesize 2-5As. Whereas chemical synthesis is cumbersome being reported in an eight-step procedure [32], enzyme-mediated synthesis is easier and done using purified OASs [14, 33–36]. The literature on how to resolve and concentrate individual 2-5A molecules from within a mixture is however limited, the best option currently available being the high-performance liquid chromatography (HPLC)-based method described by Morin *et al.* [34]. Here we present an alternative to this procedure, which can be used to obtain highly pure 2-5As and 2-5A core molecules at the milligram scale.

A variety of 2′-5′ oligo(adenylate) synthetase activity assays have been devised, which generally rely on ATP as substrate [4, 15, 37–39]. The assays have been used primarily to define and characterize the reaction products, and are not well suited to determine enzyme kinetic parameters, which are most easily achieved based on reactions generating only a single product. Also, the reaction products have mainly been resolved by HPLC using reverse-phased C_18_ columns or thin-layer chromatography with PEI-Cellulose, in which the former has been used for production purposes. Likewise, many 2-5A nuclease assays have been reported [18, 40–43]. They rely on very short 2′-5′ oligoadenylate substrates, especially the 2-5A core dimer ApA, which despite being commercially available, is not biologically relevant in the sense that oligoadenylates of a minimum of three AMP moieties (*i.e.* trimer) containing 5′ phosphate(s) are required for activation of RNase L [44]. Like above, the reaction products have been resolved by HPLC using reverse-phased C_18_ columns or by thin-layer chromatography with PEI-Cellulose.

This paper comprises an assay toolbox for the characterization and identification of nucleases and synthetases regulating the 2-5A system. A procedure for isolating 2′-5′ oligoadenylates is described, which results in high yields and purity of many 2-5As and 2-5A core molecules. The 2′-5′ oligoadenylates are used as substrates in enzyme assays for measurement of synthetase and nuclease activities. Amongst others, we present an enzyme assay using the A(pA)_3_ tetramer core and dATP as substrates in producing a A(pA)_3_pdA pentameric product. The assay requires prior isolation of the A(pA)_3_ tetramer core. We also present a synthetase assay using NAD^+^ and either dATP, dCTP, dGTP or dTTP as substrates for the OAS enzyme. This assay is well-suited to assess the broader cellular role expected of the OASs, based on the very diverse substrate specificity *in vitro*.

## Methods

### Purification of OAS1 and PDE12

Human OAS1 p42 containing an N-terminal His-tag (His-OAS1) cloned in the pET9d bacterial expression vector was a kind gift from Kineta (formerly Illumigen Biosciences).

The plasmid was transformed into the *E.coli* BL21 (DE3) strain and plated on selective LB agar (50 μg/mL ampicillin), followed by inoculation of single colonies to selective LB medium for incubation at 37 °C for 16 h at 200 rpm. At OD_600_ = 0.6, the cultures were added IPTG to a final concentration of 1 mM and left for overnight incubation at 20 °C at 200 rpm to allow protein expression. The cells were harvested by centrifugation at 7000 rpm for 20 min at 4 °C and ruptured using the B-PER Protein Extraction Reagent (0.5 mL/g dry weight, Thermo Scientific). The lysates were cleared by centrifugation at 12,000 rpm for 10 min at 4 °C and diluted four-fold in Buffer A (20 mM Na_2_PO_4_, 0.5 M NaCl, pH 7.4) before filtration (0.22 μM). Protein purification was with a HiTrap Chelating HP column (1 mL, GE Healthcare) charged with 0.1 M NiSO_4_ using an ÄKTAexplorer chromatography system (GE Healthcare). The elution was carried out in a gradient of Buffer B (20 mM Na_2_PO_4_, 0.5 M NaCl, 500 mM imidazole, pH 7.4): a step gradient from 0 to 10 % B, a linear gradient from 10 to 70 % B in 10 mL and a step gradient from 70 to 100 % B. The His-OAS1 eluted in the interval 10–70 %. Elution fractions were finally mixed with one volume of 80 % glycerol and stored at −80 °C. We reproducibly obtained ~2 mg of purified His-OAS1 per liter of *E.coli* cell culture using this procedure.

Human PDE12 stripped for the mitochondrial targeting peptide (mTP) and containing a C-terminal His-tag (PDE12ΔmTP-His) was cloned in the pTriEx-3 Neo expression vector (Novagen) allowing for bacterial, insect as well as mammalian expression. Protein expression and purification were carried out as described in [23]. Briefly, BL21 (DE3) cells transformed with the above-mentioned construct were grown in selective LB medium and the expression induced with IPTG. The cells were harvested by centrifugation, and ruptured by sonication and high-pressure homogenization. The cleared lysates were applied to Ni-NTA Superflow Cartridges (1 mL, Qiagen) using 300 mM of imidazole for elution, before fractionation of the eluates with a Mono Q HR 16/10 column (GE Healthcare) in a linear gradient of KCl to 500 mM. The fractions containing PDE12ΔmTP-His were finally pooled and diluted with one volume of 80 % glycerol before storage at −80 °C. We reproducibly obtained ~1 mg of purified PDE12ΔmTP-His per liter of *E.coli* cell culture using this procedure.

For protein analyses, aliquots of the purified His-OAS1 and PDE12ΔmTP-His recombinant proteins were heat-treated at 95 °C for 5 min in loading buffer containing β-mercaptoethanol, and then subjected to SDS-PAGE followed by staining with Coomassie Blue (Pierce).

### Cell culturing and transfection

HeLa cells grown in DMEM (Gibco BRL/Life Technology) with 10 % FBS and 1 % Penicillin/Streptomycin (Gibco BRL/Life Technology) were transfected using the PolyFect Transfection Reagent (Qiagen). Plasmids were the PDE12∆mTP-His construct in pTriEx-3 Neo, red-fluorescent protein (DsRed) also in pTriEx-3 Neo and as a control the empty pTriEx-3 Neo vector. Protein expression was carried out for 24 h, before lysis with 1 % NP40, 0.5 M CH_3_COOK added 2× Complete protease inhibitor cocktail (Roche). The lysates were cleared by centrifugation at 20,000 × *g* for 3 min, and the supernatants containing the proteins stored at −80 °C. The BCA Protein Assay Kit (Pierce) was used for determination of the protein concentrations.

### Synthesis and isolation of 2-5As and 2-5A core molecules

In 200 mL reactions, 17 μg/mL of human His-OAS1 was incubated with 2 mM ATP, 4 mM Mg(OAc)_2_, 0.2 mM DTT, 40 μM EDTA, 0.2 mg/mL Poly(I)-Poly(C), 0.1 mg/mL BSA, 2 % glycerol and 4 mM Tris-Cl pH 7.8 for 3 h at 37 °C [45]. In the time span 20–90 min, ATP was added every 10 min to sequentially increase the ATP concentration by 1 mM ending with with a final concentration of 10 mM. The reaction sample was heat-inactivated at 85 °C for 15 min before filtration (0.45 μm). The analysis of 2-5A synthesis was carried using a Mono Q HR 5/5 column (GE Healthcare) on the ÄKTAexplorer. A 200 μL aliquot was applied to the column equilibrated in Buffer C (20 mM Tris–HCl, pH 7.5) and fractionated in a linear gradient of 0–20 % and 20–22.8 % of Buffer D (1 M NaCl, 20 mM Tris–HCl pH 7.5) using 18 column volumes (CV) and 50 CV, respectively. The 200 mL reaction was then divided in two equal parts (100 mL each), one part for isolation of 2-5As and the other for isolation of 2-5A core molecules. The 2-5A core molecules were produced by incubation of the 2-5As with alkaline phosphatase (100 U/mL) for 16 h at 37 °C. Preparative fractionation of 2-5As and 2-5A core molecules were with the Mono Q HR 16/10 column using a linear gradient of 0–10 % and 10–30 % of Buffer D over 3.6 CV and 24 CV, respectively. The sample injection volume were ~10 mL for each of the fractionations, therefore the method was repeated several times in order to obtain separation of the entire reaction volume of 200 mL. For concentration of the oligoadenylates, the fractions of successive runs representing identical peaks were pooled and diluted 10 fold in Buffer C, in most cases resulting in total volumes of 1–2 l. This was applied over night to the Mono Q HR 16/10 column using the inlet A of the ÄKTAexplorer chromatography system running at a constant flow rate of 1 mL/min. The day after, elution was performed by a step gradient to 50 % of Buffer D, allowing for highly concentrated fractions of the individual oligoadenylates. Purity and concentration of the oligoadenylates were determined by application of small aliquots (~20 μl) to the Mono Q HR 16/10 column as described above. For concentration determination, ATP was used as an internal control. All the chromatographic procedures were carried using 254 nm as the absorbance wavelength. We used mass spectrometry for the exact identification of the isolated 2′-5′ oligoadenylates.

### The single-product 2′-5′ oligo(adenylate) synthetase (OAS) assays

The dATP/A(pA)_3_ assay was carried out in a 40 μl reaction volume containing 32 ng of purified recombinant human His-OAS1 protein, 2 mM dATP and 0.5 mM A(pA)_3_ in 10 mM Mg(OAc)_2_, 1 mM DTT, 0.1 mg/mL BSA, 0.2 mM EDTA, 0.4 mg/mL poly(I):poly(C), 10 % glycerol, 0.1 mg/ml Creatine Kinase, 6 mM Creatine phosphate and 20 mM Tris–HCl, pH 7.8 for 30 min at 37 °C using a thermal cycler.

The NAD^+^/dNTP assay was performed in a 40 μl reaction volume using 32 ng of purified recombinant human His-OAS1 protein, 2 mM dNTP (dATP, dCTP, dGTP or TTP, respectively) and 3 mM NAD^+^ in 10 mM Mg(OAc)_2_, 1 mM DTT, 0.1 mg/mL BSA, 0.2 mM EDTA, 0.4 mg/mL poly(I):poly(C), 10 % glycerol, 0.1 mg/ml Creatine Kinase, 6 mM Creatine phosphate and 20 mM Tris–HCl, pH 7.8 for 1 h at 37 °C using a thermal cycler.

The reactions were stopped by heat shock at 94 °C for 5 min before dilution with ddH_2_O (3.5 fold) and filtration through AcroPrep^TM^ 10 K Filter Plates (Pall Corporation) by centrifugation at 3000 x *g* for 15 min. For analysis of the samples 20 μl of filtrate was applied to a HiTrap Q column (1 mL, GE Healthcare) and fractionated with a stepwise linear gradient of 0–9 %, 9–17 %, 17–17.7 %, 17.7–22 %, 22–100 % of Buffer D in C over 3 mL, 54 mL, 20 mL, 50 mL and 6 mL, respectively. The chromatograms were obtained using 254 nm as the absorbance wavelength.

The enzyme activity reaction catalyzed by OAS can be formulated with the general formula below, in which S1 and S2 are the substrates and P the product formed:

$$ S1+S2\ \to\ P $$

E, the specific activity of the enzyme (mole P generated per second per gram protein) can be calculated from the equation:

$$ E = \frac{P}{P+S2} \times \frac{{\left[S2\right]}_{start}\times V}{T\times M} $$

[*S*2]_*start*_ is the start conc of S2, V is the reaction volume, M is the amount of protein added and T is the reaction time.

The reaction schemes for the two kinds of OAS activities measured in this paper are depicted in reaction 1 and reaction 2, respectively.

Reaction 1: *dATP* + *A*(*pA*)_3_ → *A*(*pA*)_3_*pdA*

In reaction 1, the specific activity of the OAS1 enzyme (E1(OAS1) with dATP and A(pA)_3_ as substrates (S1 and S2, respectively) was calculated with the formula:

$$ E1(OAS1)=\frac{{\displaystyle \int }A{(pA)}_3pd{A}_{254 nm}}{{\displaystyle \int }A{(pA)}_3pd{A}_{254 nm}+{\displaystyle \int }A{(pA)}_{3,254 nm}}\times \frac{{\left[A{(pA)}_3\right]}_{start} \times 0.04\ {10}^{-3}l}{1800\ s\times 32\times {10}^{-9}g} $$

The integral refers to the area under the individual peaks from the substrates and products in the chromatograms.

Reaction 2: *dNTP* + *NAD*^+^ → *NAD* − *pdN*

In reaction 2, dNTP (dATP, dCTP, dGTP or TTP) and NAD^+^ were used as substrates (S1 and S2, respectively) in reactions with the OAS1 enzyme. Here the extinction coefficients, ε (M^−1^ × cm^−1^) of the individual dNTPs at 254 nm were used in the calculation of the specific enzyme activities, *i.e.* 15.200 for dATP, 9.300 for dCTP, 13.700 for dGTP and 9.600 for TTP. Thus the OAS1 specific enzyme activities E2(OAS1) from these reactions were calculated with the formula:

$$ E2(OAS1) = \frac{\frac{{\displaystyle \int }NAD-pD{N}_{254 nm}}{\varepsilon_{NAD}+{\varepsilon}_{dNTP}}}{\frac{{\displaystyle \int }NAD-pD{N}_{254 nm}}{\varepsilon_{NAD}+{\varepsilon}_{dNTP}}+\frac{{\displaystyle \int }NA{D}_{254 nm}}{\varepsilon_{NAD}}} \times \frac{{\left[NA{D}^{+}\right]}_{start} \times 0.04 \times {10}^{-3}l}{3600\ s \times 32 \times {10}^{-9}g} $$

### The 2-5A nuclease assay

The nuclease reactions were performed in a total volume of 20 μl containing 1.2 μg of purified recombinant PDE12ΔmTP-His or 10 μg of crude protein extract together with 0.5 mM of substrate, either A(pA)_3_, A(pA)_4_ or A(pA)_5_. The reactions also contained 1 mM MgCl_2_, 1 mM DTT, 1 mg/mL BSA, 1x protease inhibitor (EDTA-free, Roche Applied Science) and 20 mM Hepes, pH 7.0. The reactions were incubated for 1 h at 37 °C using a thermal cycler, and stopped by heating to 94 °C for 5 min. The end-reactions were diluted in Buffer C (15 fold) and applied to AcroPrep 10 K Filter Plates by centrifugation at 3000 × *g* for 15 min. The filtrates were analyzed on a Mono Q HR 5/5 column on an ÄKTAexplorer using a linear gradient of 0–12 % and 12–16 % of Buffer D in C over 18 CV and 20 CV, respectively. The chromatograms were obtained with 254 nm as the absorbance wave-length.

For the PDE12 activity assay the following reaction scheme was used:

$$ \mathrm{A}{\left(\mathrm{p}\mathrm{A}\right)}_n\to \mathrm{A}{\left(\mathrm{p}\mathrm{A}\right)}_{\mathrm{m}=\mathrm{n}\hbox{-} 1} + \mathrm{A}{\left(\mathrm{p}\mathrm{A}\right)}_{\mathrm{m}=\mathrm{n}\hbox{-} 2}+\dots +\mathrm{n}\ \mathrm{x}\ \mathrm{A}\mathrm{M}\mathrm{P} $$

n ≥ 1 and m ≥ 0.

The specific activity of PDE12 (mole 5′-AMP synthesized per second per gram of protein (mole AMP/(sec*g)) was calculated with the formula:

$$ E(PDE12) = \frac{{\displaystyle \int } AM{P}_{254 nm}}{{\displaystyle \int } Tota{l}_{254 nm}} \times \frac{{\left[A{(pA)}_n\right]}_{start} \times \left(n+1\right)\ x\ V}{T \times M} $$

*Total*_*254nm*_ refers to the sum of the integral of all peaks in a given chromatogram at 254 nm for a single reaction, [A(pA)_n_]_start_ is the start concentration of the substrate A(pA)_n,_ V is the reaction volume, T the reaction time and M the amount of protein added.

As an example, the tetramer core substrate would be expected to undergo cleavage into the following products using PDE12 as the enzyme:

Reaction 3: *A*(*pA*)_3_ → *A*(*pA*)_2_ + *ApA* + *A* + 3 × *AMP*

In the case of purified protein, the specific enzyme activity of PDE12, E(PDE12) could therefore be calculated using the formula:

$$ E(PDE12)=\frac{{\displaystyle \int } AM{P}_{254 nm}}{{\displaystyle \int }A{(pA)}_{3,\ 254 nm}+{\displaystyle \int }A{(pA)}_{2,\ 254 nm}+{\displaystyle \int }Ap{A}_{254 nm}+{\displaystyle \int }{A}_{254 nm}+{\displaystyle \int } AM{P}_{254 nm}} \times \frac{{\left[A{(pA)}_3\right]}_{start} \times 4 \times 0.02 \times {10}^{-3}l}{3600\ s\ x\times 1.2\ x\ {10}^{-6}g} $$

For reactions including crude protein extracts, the specific enzyme activities were calculated in mmole AMP/(sec*g of total protein). Note that the above formulas are valid only to enzymes producing one molecule of AMP per reaction cycle, which is true for exonucleases like PDE12. No endonucleases degrading 2-5As are currently known.

## Results

### Recombinant OAS1 and PDE12 proteins

To allow production of 2-5As as well as to develop and demonstrate the enzyme assays, recombinant OAS and PDE12 proteins were expressed and purified from bacteria. Synthesis of 2-5As was performed with a human OAS1 isoform known to produce oligomers up to 30-mers *in vitro* [5]. The OAS2 and OAS3 can also produce oligomers of considerable length. OAS2 has been found to produce oligoadenylates of similar length as OAS1 [5], whereas OAS3 can produce oligoadenylates of at least 12-mer length [46, 47]. Expression and purification of OAS1 isoforms are straightforward compared with OAS2. Two splice variants, p42 and p46 are the main products of the human OAS1 gene. They are identical in their N-termini of 346 aa named the core OAS unit and differ in their C-termini with extensions of 18 and 54 aa, respectively [5]. The OAS1 variant used here is the human p42 form cloned with an N-terminal His-tag fusion (His-OAS1).

The mitochondrial localization of human PDE12 is facilitated by an N-terminal mitochondrial targeting peptide (mTP) spanning residues 1–16. For the present study, we used a His-tagged variant of PDE12 lacking the mTP (PDE12ΔmTP -His), which has previously been found not to alter the enzyme activity [23]. This variant was used primarily due to the fact that expression and purification was easier compared with the full-length form.

Briefly, recombinant human His-OAS1 p42 and PDE12ΔmTP-His were expressed using the *E.coli* BL21 (DE3) strain. Purification of OAS1 p42 was done by Ni^2+^-chromatography in one step, and purification of PDE12ΔmTP-His was done in two steps, Ni^2+^- chromatography and quaternary (Q) anion-exchange chromatography. Isolated fractions were of high homogeneity as evaluated by Coomassie Blue stained SDS-PAGE protein gels (Additional file 1: Figure S1).

### Production of 2-5As and 2-5A core molecules

**Fig. 1:**
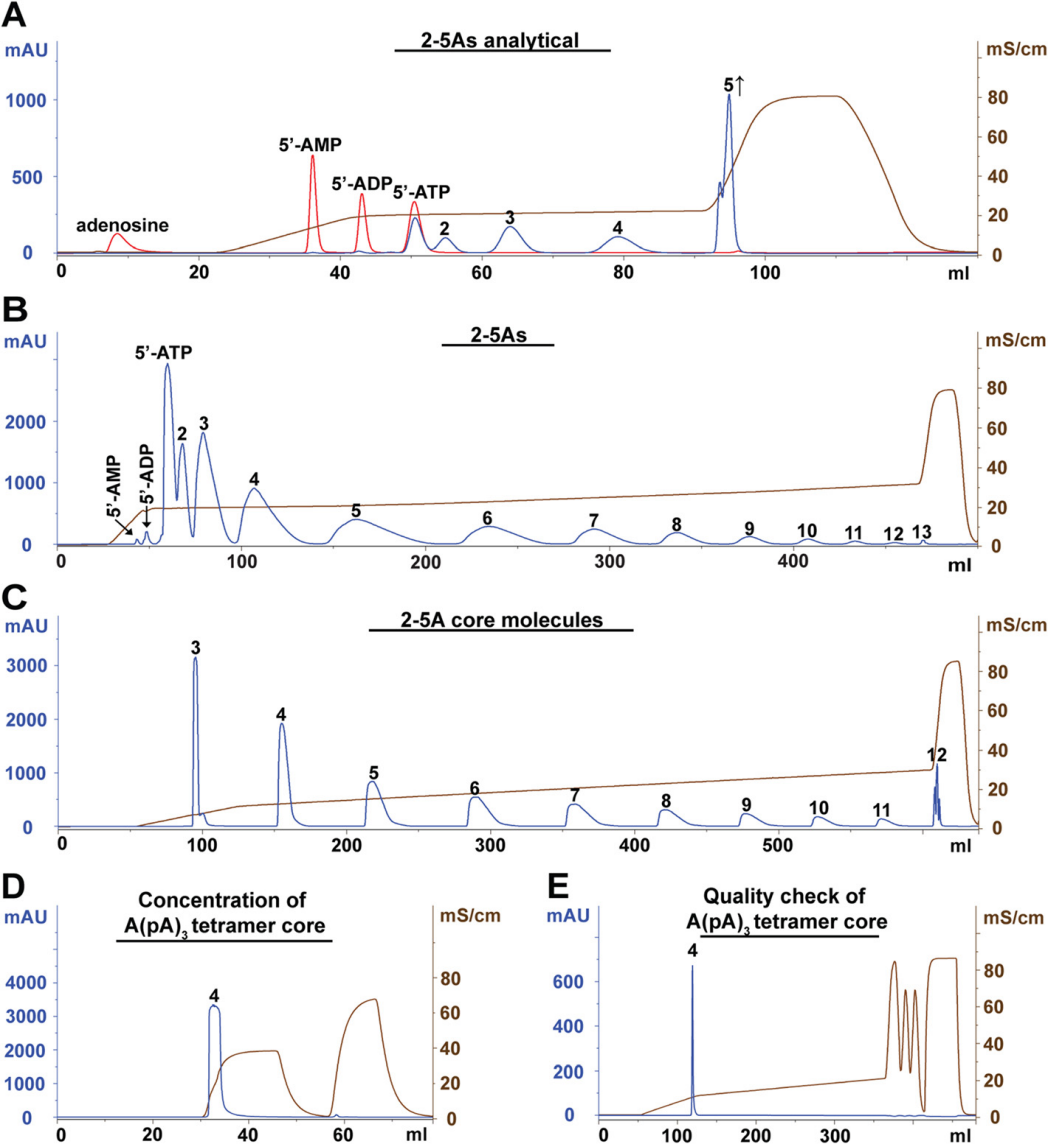
Production of single 2-5As and 2-5A core molecules. 2-5As were synthesized using human recombinant His-OAS1 protein, and subsequently resolved and concentrated by Mono Q chromatography. (**a-e**) Chromatograms representing different stages of the procedure. (**a**) Analysis of 2-5A synthesis. (**b** and **c**) Large-scale fractionation of 2-5As and 2-5A core molecules. (**d**) Example of up-concentration of a 2′-5′ oligoadenylate. In this case the A(pA)_3_ tetramer core. (**e**) Quality check of a 2′-5′ oligoadenylate (again the A(pA)_3_). Blue curves: The 2′-5′ oligoadenylates numbered according to length, hence 2 specify the pppApA (or ApA) dimer, 3 the pppA(pA)_2_ (or A(pA)_2_) trimer, 4 the pppA(pA)_3_ (or A(pA)_3_) tetramer and so forth. Red curve in (**a**): Chromatogram obtained using control molecules: Adenosine, 5′-AMP, 5′-ADP and 5′-ATP as indicated. Brown curves: Experimental salt gradients. mAU (milli-absorbance unit) is the unit used for molecular detection at 254 nm, and mS/cm (milliSiemens/centimeter) the unit used to measure conductivities

Compared with 3′-5′ phosphodiester-linked oligonucleotides, 2-5As are difficult to attain chemically. Therefore, we synthesized the 2′-5′ oligoadenylates by enzymatic means using the His-OAS1 p42 variant in large-scale reactions with ATP. The efficiency of the reaction was assessed by Q anion-exchange chromatography, and the resulting chromatogram can be seen in Fig. 1a. In this figure two chromatograms have been superimposed, the 2-5A reaction mix (blue curve) and the control molecules (red curve): adenosine, 5′-AMP, 5′-ADP and 5′-ATP, in order to visually distinguish the reaction products (2-5As) from the ATP substrate. The batch of synthesised 2-5As was subsequently split in two parts, one part for isolation of 2-5As and the other for isolation of 2-5A core molecules. The conversion from 2-5As into 2-5A core molecules was performed by treatment with alkaline phosphatase. The 2-5As and 2-5A core molecules were then resolved by Mono Q chromatography, which resulted in high quality and quantity of many individual 2-5A molecules. Fig. 1b and c show how individual 2-5As and 2-5A core molecules up to and above the decamer length were successfully resolved. A homogenous preparation of the pppApA, the 2-5A dimer could not be obtained due to co-elution with ATP (see Fig. 1b). Also, adenosine and the ApA core dimer were found to only weakly bind the resin under the experimental conditions used (running buffer: 20 mM Tris–HCl, pH 7.5). These molecules were thus detected before application of the experimental salt gradient used for separation, but after the void volume of the column. The individual 2-5As and 2-5A core molecules were subsequently concentrated in three steps, by a 10 fold dilution of the samples in running buffer to reduce the overall sodium chloride content, re-application of the molecules to the Mono Q column and elution by a step-gradient of 0.5 M sodium chloride. In Fig. 1d the elution profile obtained from concentration of the A(pA)_3_ tetramer core is shown. Here a starting volume of ~200 mL (*i.e.* before dilution) was converted to less than 3 mL (~70 fold concentration of the molecule). In Fig. 1e, an aliquot of the final concentrated sample was resolved by Mono Q chromatography to confirm molecular purity. The detection of a single peak at the expected retention volume, suggests that this a very homogenous preparation of the A(pA)_3_ tetramer core. Using this procedure, we have obtained very high yields of many individual 2′-5′ oligoadenylates. Of the three substrates utilized here: A(pA)_3_ tetramer core, A(pA)_4_ pentamer core and A(pA)_5_ hexamer core, a total of 17.2, 15.5 and 11.3 mg were obtained corresponding to 10.1, 9.1 and 6.6 mg of oligoadenylate per mg human His-OAS1 used in the reaction, respectively.

### The single-product OAS activity assays

We have developed a number of OAS enzyme assays using the isolated oligoadenylates as well as NAD^+^ as substrates. The assays produce only a single reaction product, as opposed to the large panel of 2-5As formed using ATP as substrate. This allows for a more precise kinetic assessment of the enzymes.

In the first assay, the A(pA)_3_ tetramer core and the deoxy-nucleotide dATP were used as substrates in formation of a pentameric product, A(pA)_3_pdA. Because dATP, as opposed to ATP is blocked for elongation/acceptance of nucleotides due to an inert 2′ hydrogen atom, the dATP will only function in the donor-site of the enzymes. Similarly, the A(pA)_3_ will be committed to the acceptor-site due to a lack of 5′ phosphates. Consequently, the single A(pA)_3_pdA product formed is blocked for further 2′ elongation by incorporation of dAMP. The reaction can be summarized as follows: *dATP + A(pA)*_*3*_ *→ A(pA)*_*3*_*pdA + PPi*. The fact that all reactants and products are negatively charged at physiological pH allow for fractionation by anion-exchange chromatography.

**Fig. 2:**
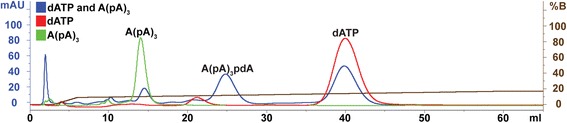
Single-product OAS assay using dATP and A(pA)_3_ as substrates. Purified human recombinant His-OAS1 enzyme was incubated with dATP and A(pA)_3_ for 30 min at 37 °C, and the reactants and product resolved using a HiTrap Q column. In the figure, three separate chromatograms have been superimposed with the position of the respective molecules indicated. Blue curve: Profile from a reaction using His-OAS1 with dATP and A(pA)_3_ as substrates. Red and green curves: Control reactions using His-OAS1 together with dATP or A(pA)_3_ alone, respectively. Brown curve: Experimental salt gradient. Chromatograms were obtained with 254 nm as the absorbance wavelength. mAU, milli-absorbance unit and mS/cm, milliSiemens/centimeter

For demonstrating the assay we incubated the purified human His-OAS1 with the substrates dATP and A(pA)_3_ and allowed the reaction to proceed for 30 min. The reactants and products were resolved by anion-exchange chromatography using a HiTrap Q column and a gradient of increasing sodium chloride concentration. Compared with the controls dATP or A(pA)_3_ only, the product A(pA)_3_pdA exhibited a retention volume higher than A(pA)_3_ and lower than dATP, and formed a discrete peak (Fig. 2). We calculated the specific enzyme activity of the human OAS1 p42 for production of A(pA)_3_pdA to be 1.1 mmole/(sec*g).

**Fig. 3:**
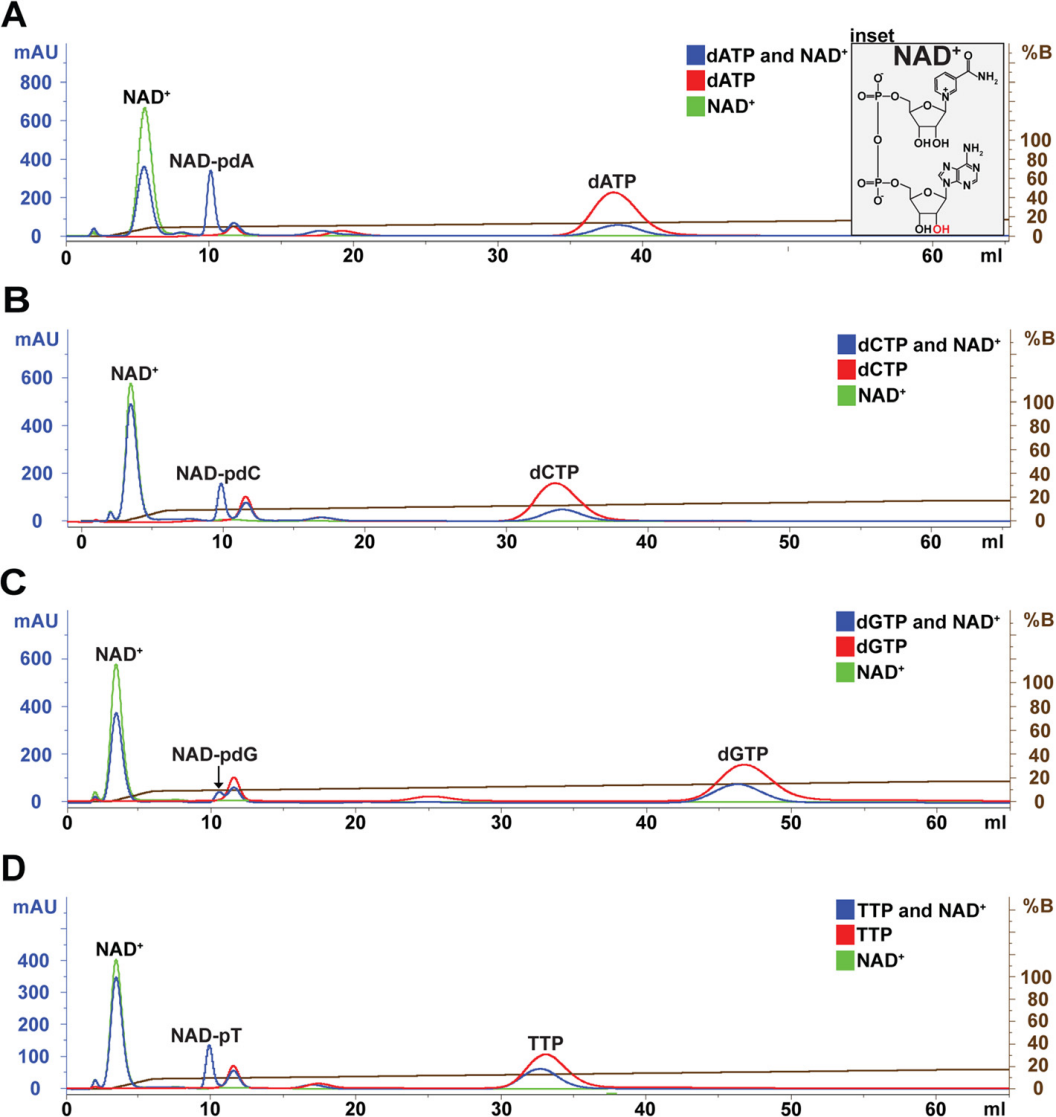
Single-product OAS assay using dNTPs and NAD^+^ as substrates. The purified human recombinant His-OAS1 enzyme was incubated with NAD^+^ and dNTP (dATP, dCTP, dGTP or TTP) for 30 min at 37 °C, and the reactants and product resolved using a HiTrap Q column. Each figure (**a-d**) includes three chromatograms that have been superimposed. The position of the reactants and product are indicated in the chromatograms. The substrates used were: (**a**) NAD^+^ and dATP, (**b**) NAD^+^ and dCTP, (**c**) NAD^+^ and dGTP and (**d**) NAD^+^ and TTP. Blue curves: Profiles from reactions using His-OAS1 together with NAD^+^ and dNTP. Red and green curves: Control reactions after incubation of His-OAS1 with either NAD^+^ or dNTP alone, respectively. The insert in (**a**) shows the structural formula of NAD^+^. The 2′ hydroxyl (OH) to be linked with dNMP during the reaction has been highlighted in red. Brown curves: experimental salt gradients. Chromatograms were obtained by using 254 nm as the absorbance wavelength. mAU, milli-absorbance unit and mS/cm, milliSiemens/centimeter

We also made additional set-ups and optimized the assay using alternative ‘acceptor’ and ‘donor’ molecules as substrates for the OAS (Fig. 3). NAD^+^, a coenzyme present in all living cells is composed of two nucleotides one containing an adenine base (the 2′ nucleotide) and the other a nicotinamide base (Fig. 3a, inset). Both in terms of unavailability of free/reactive 5′ phosphates and the presence of a 2′ hydroxyl group, NAD^+^ closely mimics the A(pA)_3_ substrate. Consequently, we substituted the A(pA)_3_ substrate with NAD^+^ and carried out the reactions with all of the dNTPs (dATP, dCTP, dGTP and TTP) separately, which should allow for synthesis of single-product NAD-dNMP derivatives. Indeed the human His-OAS1 was found to produce NAD-dNMP derivatives albeit with variable efficiencies, resulting in nice and discrete peaks with retention volumes higher than NAD^+^ and lower than the dNTPs (Fig. 3). The specific enzyme activities were calculated to be 0.2 mmole/(sec*g) for NAD-pdA; 0.1 mmole/(sec*g) for NAD-pdC; 0.02 mmole/(sec*g) for NAD-pdG and 0.1 mmole/(sec*g) for NAD-pT.

### The 2-5A nuclease assay

**Fig. 4:**
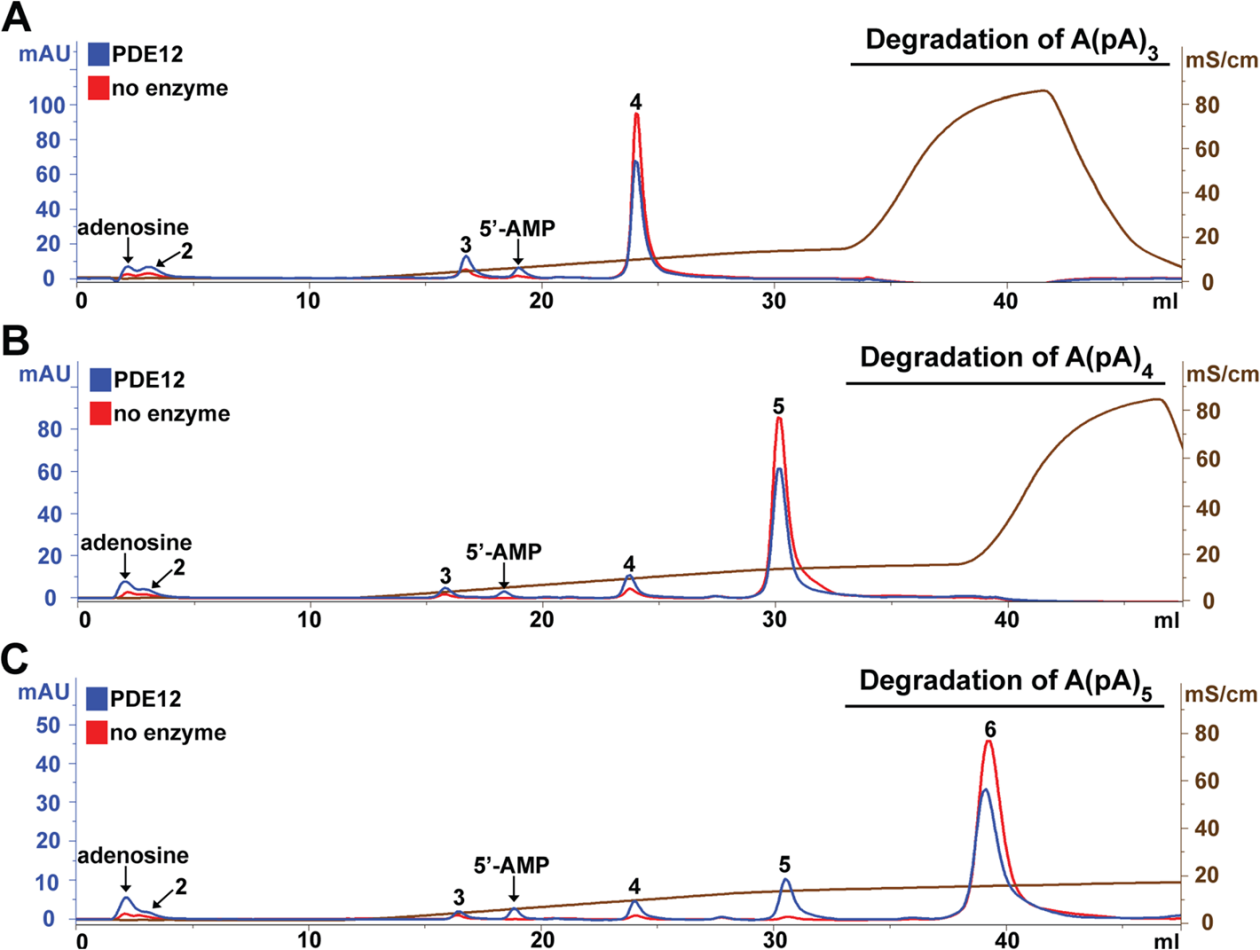
2-5A nuclease assay using a purified protein. 1.2 μg of purified human recombinant PDE12ΔmTP-His enzyme was incubated in reactions with (**a**) A(pA)_3_ tetramer core, (**b**) A(pA)_4_ pentamer core or (**c**) the A(pA)_5_ hexamer core for 1 h at 37 °C, and the products resolved by Mono Q chromatography. Each figure (**a-c**) includes two chromatograms that were superimposed. Blue curves: Profiles from reactions in which the enzyme was used, and red curves: Profiles from reactions where the enzyme was omitted. The 2-5A core molecules were numbered according to length with 2 specifying the ApA dimer core, 3 the A(pA)_2_ trimer core and so forth. Brown curves: Experimental salt gradients. Chromatograms were obtained with 254 nm as the absorbance wavelength. mAU, milli-absorbance unit and mS/cm, milliSiemens/centimeter. This figure has been reproduced from [19] with courtesy of Biochimie, Elsevier

**Fig. 5:**
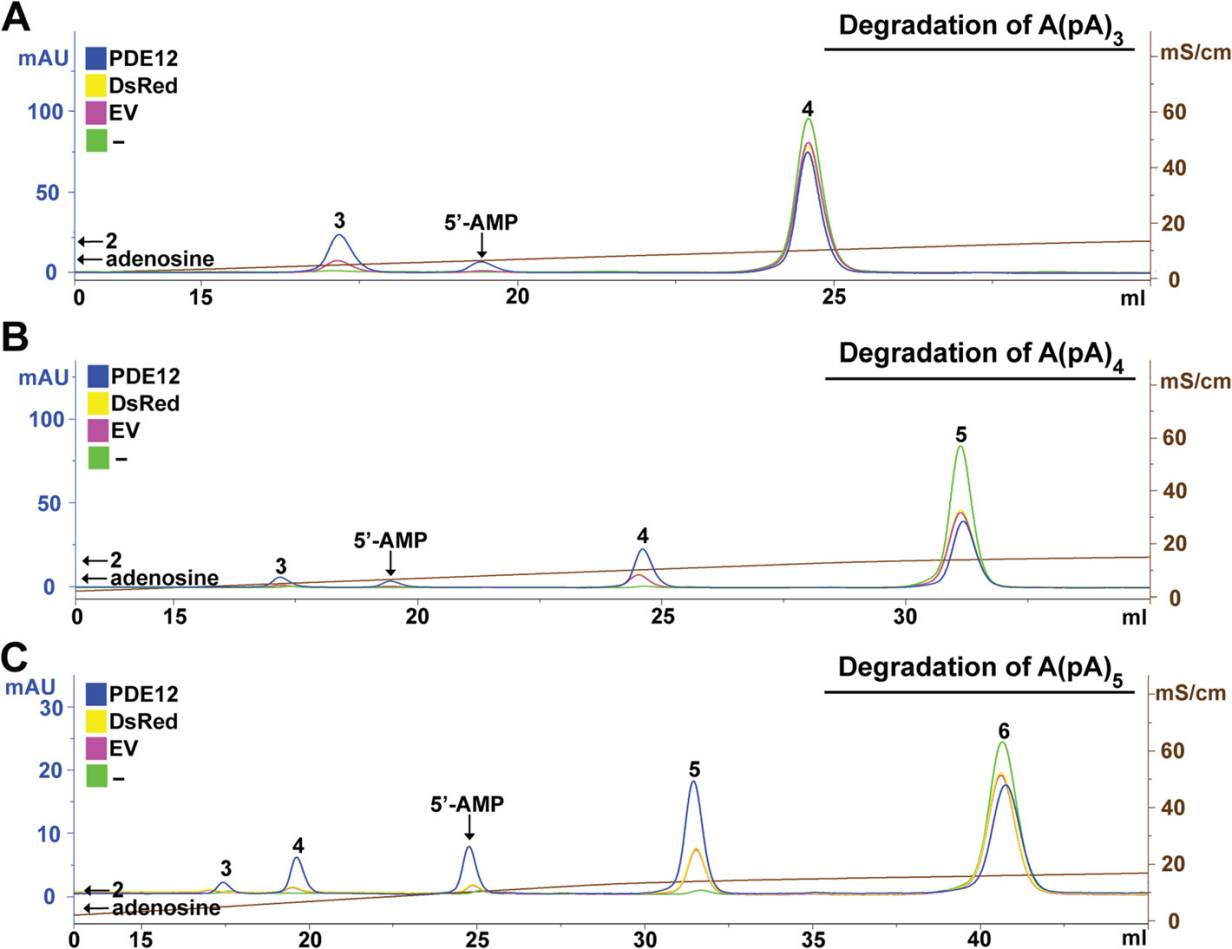
2-5A nuclease assay using crude protein extracts. 10 μg of crude protein extracts of HeLa cells transfected with PDE12ΔmTP-His, red-fluorescent protein (DsRed), empty-vector pTriEx-3 Neo (EV) or mock-transfected (−) were incubated with (**a**) A(pA)_3_ tetramer core, (**b**) A(pA)_4_ pentamer core or (**c**) A(pA)_5_ hexamer core for one hour at 37 °C, and the products resolved by Mono Q chromatography. Note that each figure (**a-c**) includes four superimposed chromatograms: blue curves PDE12, yellow curves DsRed, purple curves EV and green curves -. The 2-5A core molecules are numbered according to length with 2 specifying the ApA dimer core, 3 the A(pA)_2_ trimer core and so forth. Brown curves: Experimental salt gradients. The chromatograms were obtained with 254 nm as the absorbance wavelength. mAU, milli-absorbance unit and mS/cm, milliSiemens/centimeter

To be able to better identify and characterize nucleases regulating cellular 2-5A levels we have developed a simple 2′-5′ nuclease assay. The reactions were set up using a single 2′-5′ oligoadenylate substrate, which are combined with a (candidate) nuclease and incubated for a given amount of time. The assays demonstrated here utilize the tetramer core A(pA)_3_, pentamer core A(pA)_4_ and hexamer core A(pA)_5_ substrates in reactions with the purified PDE12ΔmTP-His enzyme or HeLa cell protein extracts. The substrate and products were resolved by Mono Q chromatography. The chromatograms for purified protein (PDE12ΔmTP-His) are shown in Fig. 4 and for HeLa cell protein extracts in Fig. 5. Different controls were included. For the purified protein, a reaction omitting the enzyme was used to measure the extent to which the substrate might undergo autohydrolysis. For the crude protein extracts, the contribution of normal cellular proteins to total enzyme activities, *i.e.* endogenous activities versus total enzyme activities, need to be distinguished to precisely map the activity specific to the expressed recombinant protein. This was done by including a reaction of protein extracts from empty-vector (EV) transfected cells, and using this as the measure of cellular endogenous activity (purple curves in Fig. 5). We also carried out a reaction with protein extracts from HeLa cells overexpressing an irrelevant protein (dsRed), to ascertain if substrate degradation is specific to PDE12, or simply is the result of overexpressing a protein (DsRed, yellow curves in Fig. 5). Please note that the activity chromatograms representing the EV (purple) and DsRed (yellow) controls in Fig. 5 closely overlap (as expected), and thus cannot be discerned in the figure.

The levels of high molecular weight products are higher than low molecular weight products, *e.g.* for degradation of A(pA)_5_ hexamer core the level of products are: pentamer core > tetramer core > trimer core > dimer core > adenosine (Figs. 4 and 5). This correlates with the fact that PDE12 has been described as a 2′-5′ exonuclease producing 5′-AMP during each reaction cycle, which is temporally separated by dissociation and re-binding of substrates (*i.e.* a distributive enzyme) [19]. The distributive cleavage of 2′-5′ oligoadenylates by PDE12 can be described with a single cleavage event conforming to the reaction scheme: *(p)*_*x*_*A(pA)*_*n*_ *→ (p)*_*x*_*A(pA)*_*n-1*_ *+ AMP*, where x = [0, 1, 2 or 3], n ≥ 1, implying that substrates can be processed until n = 1. Processive enzymes on the other hand might catalyze the reactions to completion generating two reaction products exclusively: adenosine plus AMP for 2-5A core molecules and ATP plus AMP for 2-5As. The following reaction scheme describes this mode of catalysis: *(p)*_*x*_*A(pA)*_*n*_ *→ (p)*_*x*_*A + nAMP,* where x = [0, 1, 2 or 3] and n ≥ 1. Note that regardless of the degradation mechanism (distributive versus processive), one molecule of AMP is produced per cleavage event assuming that degradation is mediated by an exonuclease activity. Therefore the activities obtained from different nucleases on a single substrate as well as for one nuclease on several substrates, are directly comparable provided that the conclusions will be based on the quantity of AMP generated. Table 1 provides an overview of the specific enzyme activities calculated for PDE12, based on the reactions presented. Note that for crude protein extracts, the specific activity attributable to PDE12 only, can be inferred by subtraction of the cellular endogenous activities (either DsRed or EV analyses) from total enzyme activities (PDE12 reactions).

**Table 1:** Specific enzyme activities of PDE12

	**Purified protein**			
	(10^−3^ mmole 5′-AMP/(sec*g))			
Substrate	PDE12		no enzyme	
A(pA)_3_	6.1 ± 0.3		0.2 ± 0.1	
A(pA)_4_	6.6 ± 0.5		0.1 ± 0.2	
A(pA)_5_	6.9 ± 0.6		0.0 ± 0.2	
	**Crude extracts**			
	(10^−3^ mmole 5′-AMP/(sec*g))			
Substrate	PDE12	DsRed	EV	-
A(pA)_3_	2.2 ± 0.2	0.8 ± 0.2	0.6 ± 0.4	0.1 ± 0.2
A(pA)_4_	2.0 ± 0.4	0.6 ± 0.3	0.4 ± 0.5	0.2 ± 0.1
A(pA)_5_	1.7 ± 0.3	0.4 ± 0.4	0.7 ± 0.3	0.0 ± 0.2

Purified human PDE12ΔmTP-His or PDE12ΔmTP-His in HeLa cell protein extracts were incubated for 1 h at 37 °C in reactions with A(pA)_3,_ A(pA)_4_ or)pA)_5_as substrates. The specific enzyme activities were calculated based on the chromatographic elution profiles, using the formulas outlined in the methods section. DsRed, EV and -: extracts from HeLa cells transfected with a construct encoding red-fluorescent protein, the empty-vector of pTriEx-3 Neo or mock-transfected, respectively. no enzyme: control reaction in which PDE12ΔmTP-His was omitted from the reaction. The data are provided as mean values of three replicates with ± values listing the standard error of the mean (s.e.m.)

## Discussion

This paper presents a toolbox for characterization and identification of the nucleases and synthetases regulating cellular 2-5A levels. A procedure for isolating 2-5A molecules is described along with enzyme assays using purified OAS and PDE12 recombinant enzymes.

### The single-product OAS activity assays

Measurements of OAS enzyme activity have traditionally been assayed using ATP as substrate producing a mixture of 2-5A molecules [4, 15, 37, 39]. Our assays produce only a single reaction product allowing a precise kinetic assessment of the enzymes. In the dATP/A(pA)_3_ assay, human OAS1 p42 was found to produce the A(pA)_3_pdA pentamer product with a specific activity of 1.1 mmole/(sec*g). An estimate of 0.2 mmole/(sec*g) on formation of 2-5As using ATP as substrate has previously been determined for human OAS1 p42 [48]. This difference (5–6 fold) might result from the lower estimate being based on formation of multiple products, *i.e.* 2-5As compared with the single product A(pA)_3_pdA formed in our assay. Variations may also result from the approach used to quantify reactants and products. We carried out quantification based on the integral of the corresponding peaks following anion-exchange chromatography, whereas in [48] quantification was based on the densitometric scans of dots (molecules) resolved by thin-layer chromatography.

In the dNTP/NAD^+^ assay, human OAS1 p42 was found to produce NAD-dNMP derivatives with specific activities of 0.2 mmole/(sec*g) for NAD-pdA, 0.1 mmole/(sec*g) for NAD-pdC, 0.02 mmole/(sec*g) for NAD-pdG and 0.1 mmole/(sec*g) for NAD-pT. Similar assays using rabbit OAS1 in reactions with NAD^+^ and NTPs (ATP, GTP, UTP and CTP, respectively) have previously been conducted yielding specific activities of 0.0009 mmole/(sec*g) for NAD-pA, 0.0005 mmole/(sec*g) for NAD-pG, 0.0009 mmole/(sec*g) for NAD-pU and 0.0008 mmole/(sec*g) for NAD-pC [15]. Indeed our estimates are higher, which may reflect the different sources of OAS used, *i.e.* human versus rabbit, the strategy undertaken for analyzing product formation, *i.e.* anion-exchange chromatography versus thin-layer chromatography, or simply that we have used dNTPs over NTPs in the reactions. Note that NAD^+^ was initially demonstrated to be a preferred primer over ATP for the OASs, producing mainly NAD^+^ derivatives such as NAD-pA in reactions of both substrates [49]. This result was however contradicted by Ferbus *et al.* [15], showing that NAD^+^ and ATP are used with equal efficiencies when part of the same reaction. In the dNTP/NAD^+^ assay demonstrated here, we chose to substitute the ATP with dNTPs to accommodate the need for a single-product assay. Importantly, the above synthetase assays are applicable also to crude extracts, *e.g.* bacterial or mammalian cell protein extracts (data not shown). This is possible through use of a creatine kinase system applied in the reaction buffer, which continuously regenerates dATP from the dADP and dAMP inhibitors formed during the reaction.

The OASs have been found to join a variety of oligoadenylates and derivatives *in vitro*. For instance, using NAD^+^, ADP-ribose and (A5′)p_4_(5′A) in reactions with ATP, the enzymes readily produce NAD-pA(pA)_2_, ADP-ribose-pA(pA)_2_ and (A5′)p_4_(5′A)-pA(pA)_2_ conjugates, respectively [49]. The varied set of products produced by the OASs *in vitro* therefore naturally raises the question if the OASs might retain other biological functions than that related to the 2-5A system. This has been studied for the NAD^+^, ADP-ribose and (A5′)p_4_(5′A) derivatives [49]. The conjugates were firstly screened for an ability to inhibit protein synthesis in an encephalomyocarditis-RNA-programmed tumour cell-free system. Whereas the (A5′)p_4_(5′A)-pA(pA)_2_ strongly inhibited protein synthesis at concentrations similar to the pppA(pA)_2_ trimer, no effect was observed for the NAD^+^ and ADP-ribose derivatives. The (A5′)p_4_(5′A)-pA(pA)_2_ could also activate RNase L in a rabbit reticulocyte lysate, which was not the case for the other derivatives. Conversion of the (A5′)p_4_(5′A)-pA(pA)_2_ conjugate to the pppA(pA)_2_ trimer was proposed as a possible way for activation of RNase L in the lysate, thereby also explaining the observation of inhibition of protein synthesis. Whether this is true, or the (A5′)p_4_(5′A)-pA(pA)_2_ conjugate inhibited protein synthesis by another mechanism, was not determined. Importantly, neither of the conjugates were detected naturally in mouse L cells even after IFN-stimulation and virus infection. This suggests that these conjugates may have functions not related to the 2-5A system and might be triggered by other stimuli.

### The 2-5A nuclease assay

Enzyme measurements of 2-5A nucleases have mainly relied on short substrates unable to activate RNase L, which requires oligoadenylates of trimer length and above [18, 40–44]. We used oligoadenylates of tetramer length and above in our assays, thereby increasing the significance with regard to the 2-5A system. Furthermore, the assay was optimized to resolve all the reactants and products by anion-exchange chromatography, which allows the direct usage of the peak integrals for calculation of enzyme activities. The activities measured for purified human PDE12ΔmTP-His and PDE12ΔmTP-His in HeLa cell protein extracts are listed in Table 1. The specific enzyme activity of PDE12 has previously been determined [41, 50]. The mouse enzyme was found to exhibit a specific activity of ~20*10^−3^ mmole/(sec*g) of 5′-AMP using the ApA dimer as substrate, and the bovine enzyme an activity of ~0.02*10^−3^ mmole/(sec*g) of 5′-AMP using the A(pA)_2_ trimer as substrate. Our values are very close to that of the mouse enzyme however differ with one to three orders of magnitude from the bovine enzyme. The fact that PDE12 is conserved throughout the mammalian lineage (*e.g.* human and bovine proteins share 92 % similarity at the amino acid level), immediately suggests that assay-dependent factors and not variation in protein sequence may have caused the observed difference [18]. For instance, the purified bovine sample appeared very crude as analyzed by SDS-PAGE silver staining, whereas ours was nearly free of contaminants. Accordingly, such presence of contaminants would inevitable lower the overall enzyme activities by a factor proportional to the amount of impurities versus PDE12 protein in the sample.

### Production of single 2′-5′ oligoadenylates

The described procedure has been used for isolation of 2-5As and 2-5A core molecules up to and above decamer length. We obtained 17.2, 15.5 and 11.3 mg of the A(pA)_3_ tetramer core, A(pA)_4_ pentamer core and A(pA)_5_ hexamer core starting from a 100 mL reaction volume, corresponding to 10.1, 9.1 and 6.6 mg of oligoadenylate synthesized per mg of human His-OAS1 used, respectively. In comparison, Morin *et al.* [34] obtained 0.27, 1.01, 0.43, 0.09 and 0.01 mg of the pppA(pA) dimer, pppA(pA)_2_ trimer, pppA(pA)_3_ tetramer, pppA(pA)_4_ pentamer and pppA(pA)_5_ hexamer in their HPLC-based method starting from a 1 mL reaction volume, respectively. The porcine OAS1 used by them exhibited a conversion rate of 33.5, 124.9, 53.5, 10.7 and 1.4 mg of oligoadenylate production per mg OAS1, respectively. Our enzyme produced higher yields of the longer oligomers (6.6 mg vs. 1.4 mg pr mg enzyme of hexamers), whereas the enzymes used by Morin *et al.* produced higher yields of the smaller oligomers (53.5 mg vs. 17.2 mg pr. mg enzyme of tetramers). Nevertheless, the total yields obtained by us are at least an order of magnitude higher, which can be ascribed to the large difference in reaction volume, *i.e.* 1 mL in Morin *et al.* [34] versus 100 mL in our case. The larger reaction volume are practical only because of the ion-exchange procedure used, where in practice, no limits exist for the sample injection volume.

### The 2-5A system

In Poulsen *et al.* [23], we described a model accounting for activation and deactivation of the 2-5A system following viral exposure based on tight regulation of 2-5As. *The OASs are induced and activated by IFNs leading to nanomolar levels of 2-5As, which activate RNase L. Unknown 5′-phosphatases convert cytotoxic 2-5As to less cytotoxic 2-5A core molecules simultaneously with viral RNA degradation by 2-5A-activated RNase L. The 2-5A core molecules finally accumulate to micromolar levels for transportation (or diffusion) by a yet to be defined mechanism to the mitochondrial matrix for degradation by PDE12.* With the recent discovery of novel cellular nucleases (ENPP1 and AKAP7) as well as virally encoded nucleases (ns2 and VP3), this process however might prove to be more complex than hitherto expected (readers are referred to a recent review by Silverman *et al.* [51]). Despite PDE12, ENPP1 and AKAP7 degrades 2′-5′ oligoadenylates *in vitro* their importance during viral infection, has yet to be reported. Consequently, wide uncertainty exists as to *i)* which enzymes take part in 2-5A degradation in cells, *ii)* what are the contribution offered by each of the enzymes to the process, *iii)* do the enzymes have a preferred cellular substrate and *iv)* what are the interplay between cellular and viral nucleases during infection. The 2-5A nuclease assay demonstrated here is suitable for identification and characterization of enzyme candidates involved in the cellular degradation of 2′-5′ oligoadenylates. The preferred substrates of the enzymes, *i.e.* 2-5As versus 2-5A core molecules can also be deduced to hypothesize the exact cellular role of the proteins in question. The assay should also work for exploration of putative 5′-phosphatases converting 2-5As to 2-5A core molecules using a 2-5A molecule as substrate. 2-5As and 2-5A core molecules are easily resolved by ion-exchange chromatography, making this assay straightforward.

The single-product dATP/A(pA)_3_ assay can be used to precisely map and characterize the activities of the individual OAS isoforms. Based on cellular localization, one could then assign distinct roles to the individual forms in the pathway. The single-product dNTP/NAD^+^ assay (or modifications hereof) could be used to more broadly address properties of enzymes exhibiting 2′-5′-nucleotidyl transferase activity. Although the 2-5A system has only been described in vertebrates, orthologues of OAS exist throughout the evolutionary tree of life [38, 52]. These enzymes might be expected to fulfill other cellular roles from that of the 2-5A system. This is in line with the diverse substrate specificity identified for the mammalian OASs, which may be a reminiscence of the evolutionary past [15].

Here we have described some enzyme assays suitable for identification and characterization of the synthetases and nucleases regulating cellular 2-5A levels. The assays might help take our understanding of the 2-5A system one step further, by serving as a platform upon which new discoveries can be made.

## Conclusions

In this paper, we have described assays for the synthetases and nucleases regulating cellular 2-5A levels including a procedure to obtain high levels of individual 2-5As and 2-5A core molecules. The synthetase assays were designed to produce a single reaction product only, allowing for precise kinetic assessment of the enzymes. Contrary, the nuclease assays produced a mixture of 2-5A molecules, which were resolved by chromatography. Our assays are well-suited for scientists aiming to identify and characterize the synthetases and nucleases regulating cellular 2-5A levels, but can also be used to address a broader cellular role of the enzymes by means of alternative substrates like dNTP and NAD^+^.

## Additional file

Additional file 1: Figure S1. OAS1 and PDE12 recombinant proteins. Analysis of purified recombinant proteins by SDS-PAGE and Coomassie Blue staining.

## Abbreviations

OASs: 2′-5′ oligoadenylate synthetases
PDE12: Phosphodiesterase 12
2-5As: 5′-triphosphorylated, 2′-5′-linked oligoadenylate polyribonucleotides
2-5A core molecules: 5′-dephosphorylated forms of 2-5As
ENPP1: Ectonucleotide pyrophosphatase/phosphodiesterase 1
AKAP7: A-kinase anchoring protein 7
NAD^+^: Nicotinamide adenine dinucleotide
