# Supplementary figures and images for: Enzyme assays for synthesis and degradation of 2-5As and other 2′-5′ oligonucleotides
Source: BMC Biochem. 2015 Jun 26;16:15. doi: 10.1186/s12858-015-0043-8 (PMC4481073; doi:10.1186/s12858-015-0043-8)

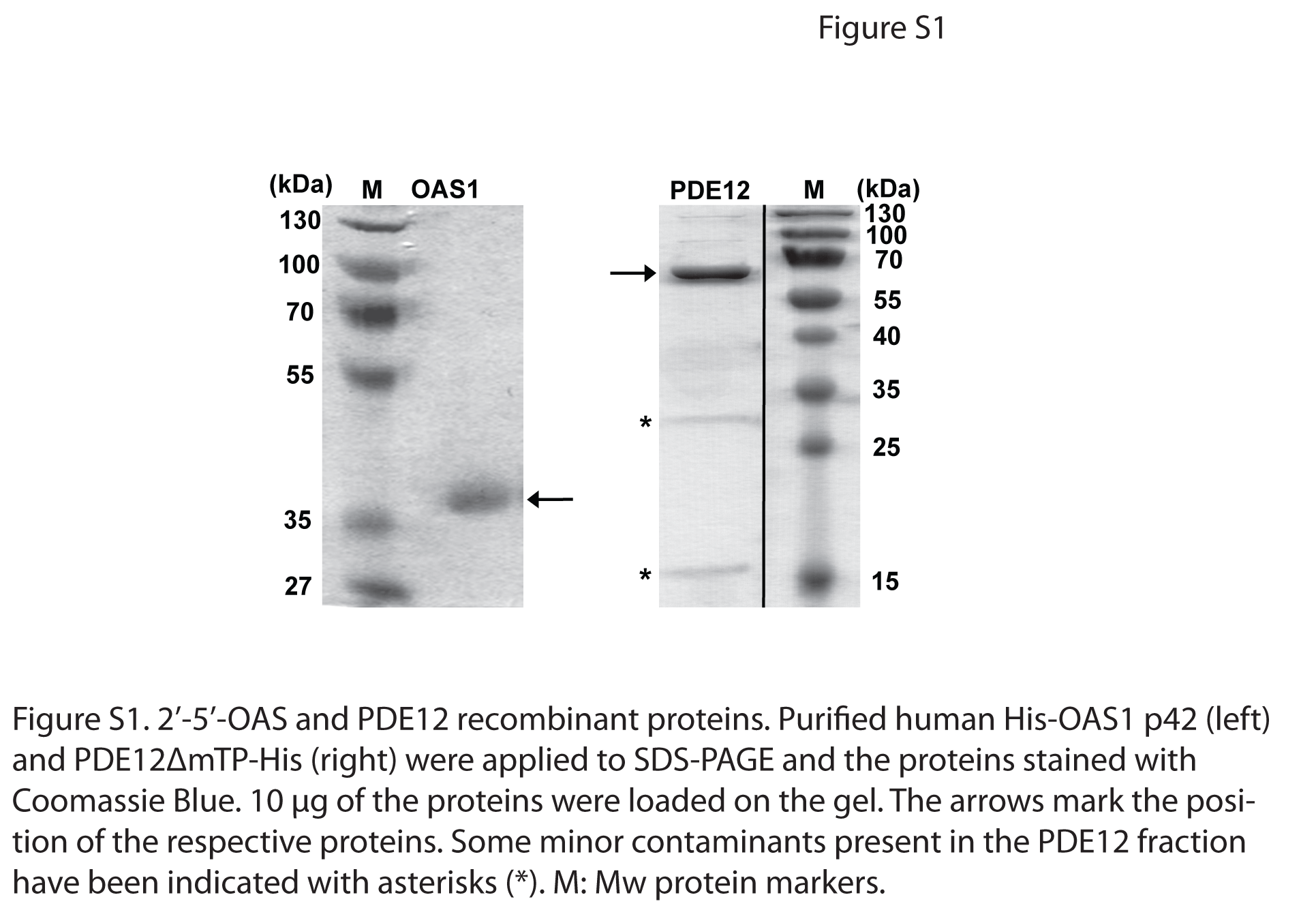

Supplement: Additional file 1: Figure S1. — OAS1 and PDE12 recombinant proteins. Analysis of purified recombinant proteins by SDS-PAGE and Coomassie Blue staining. [file 12858_2015_43_MOESM1_ESM.tif]
